# Non‐trophic impacts from white sharks complicate population recovery for sea otters

**DOI:** 10.1002/ece3.5209

**Published:** 2019-04-30

**Authors:** Jerry H. Moxley, Teri E. Nicholson, Kyle S. Van Houtan, Salvador J. Jorgensen

**Affiliations:** ^1^ Monterey Bay Aquarium Monterey California; ^2^ Nicholas School of the Environment Duke University Durham North Carolina

**Keywords:** community structure, complex interactions, nonconsumptive effects, prey mistargeting, protected species

## Abstract

Complex interactions between protected populations may challenge the recovery of whole ecosystems. In California, white sharks (*Carcharodon carcharias*) mistargeting southern sea otters (*Enhydra lutris nereis*) are an emergent impact to sea otter recovery, inhibiting the broader ecosystem restoration sea otters might provide. Here, we integrate and analyze tracking and stranding data to compare the phenology of interactions between white sharks and their targeted prey (elephant seals, *Mirounga angustirostris*) with those of mistargeted prey (sea otters, humans). Pronounced seasonal peaks in shark bites to otters and humans overlap in the late boreal summer, immediately before the annual adult white shark migration to elephant seal rookeries. From 1997 to 2017, the seasonal period when sharks bite otters expanded from 2 to 8 months of the year and occurred primarily in regions where kelp cover declined. Immature and male otters, demographics most associated with range expansion, were disproportionately impacted. While sea otters are understood to play a keystone role in kelp forests, recent ecosystem shifts are revealing unprecedented bottom‐up and top‐down interactions. Such shifts challenge ecosystem management programs that rely on static models of species interactions.

## 1 INTRODUCTION

Unexpected conservation challenges include conflict between protected populations (Marshall, Stier, Samhouri, Kelly, & Ward, 2016; Soulé, Estes, Berger, & Rio, 2003). Recovering carnivores, for example, can increase direct and indirect top‐down effects (Stier et al., 2016) that may inhibit the recovery of protected prey species and compete against larger goals of ecosystem recovery (Marshall et al., 2016; Samhouri et al., 2017). Such events, though complicated, can perhaps be anticipated from a previous empirical knowledge base of ecological community structure. More challenging to manage, however, are unprecedented interactions between protected species that have only recently arisen, and did not contribute to the historical population declines themselves. Such complex dynamics may increasingly become common as ecosystems are transformed and disrupted by climate change (Carnicer et al., 2011; Moritz et al., 2012; Sheridan & Bickford, 2011).

Protected white sharks (*Carcharodon carcharias*) and pinnipeds have an established predator–prey relationship along the California coast. White sharks are considered threatened by the IUCN Red List, and though they are protected at state, federal, and global scales, their current status in the northeastern Pacific is debated (Burgess et al., 2014; Chapple et al., 2011; Dewar, Domeier, & Nasby‐Lucas, 2004; Lowe et al., 2012). Seals and sea lions are protected taxonomically under the Marine Mammal Protection Act (“MMPA”) and many species are recovering from near‐extinction in the early 20th century and now reoccupy the full extent of their historical range (Lowry et al., 2014; Lowry, Melin, & Laake, 2017; Magera, Flemming, Kaschner, Christensen, & Lotze, 2013; Roman, Dunphy‐Daly, Johnston, & Read, 2015). In addition to protections under the MMPA, southern sea otters (*Enhydra lutris nereus*) are also protected under the International Fur Treaty of 1911 and the U.S. Endangered Species Act. Sea otters, however, have been slower to recover than pinnipeds and have reoccupied only 13% of their prior range (USFWS, 2015). Though the original causes of the sea otter population decline (hunting, fishery interactions) have been solved, the recent advent of mortalities from white shark bites suggests new threats to recovery (Nicholson et al., 2018; Tinker, Hatfield, Harris, & Ames, 2016).

Seasonal increases in elephant seal (*Mirounga angustirostris*) abundance at haul out sites along the California coast provide discrete foraging opportunities and help define the ontogenetic shifts and migratory patterns in white sharks (Brown, Lee, Bradley, & Anderson, 2010; Jorgensen et al., 2019; Pyle, Klimley, Anderson, & Henderson, 1996). At birth white sharks are piscivorous and are largely restricted to warm, nearshore areas in the Southern California Bight and northern Baja California (Dewar et al., 2004; Lowe et al., 2012; Weng et al., 2007). An ontogenetic shift in the shark's preferred habitat and diet occurs at 3–4 years of age as juvenile sharks transition to the cooler waters of central California (Weng et al., 2007)—within the sea otter's range. Following this transition, white sharks are more commonly observed visiting coastal aggregations near elephant seal colonies in central California, (Kanive et al., 2015; Klimley, 1985) and Guadalupe Island, Mexico (Domeier & Nasby‐Lucas, 2008; Hoyos‐Padilla, Klimley, Galván‐Magaña, & Antoniou, 2016). The heightened metabolic demands of endothermy (Carey et al., 1982) drive white shark prey preferences (Curtis et al., 2006; Klimley, Pyle, & Anderson, 1996). Elephant seals are a favored prey of adult white sharks due to their significant energy stores of blubber (Brown et al., 2010; Stephens, Boyd, McNamara, & Houston, 2009). By contrast, sea otters insulate themselves with a thick fur coat and provide little comparative caloric value for feeding white sharks.

Though it is increasingly clear that shark bites on otters are incidental and nonconsumptive, the emerging impact of this dynamic is only recently explored. To begin, four studies (Ames, Geibel, Wendell, & Pattison, 1996; Ames & Morejohn, 1980; Nicholson et al., 2018; Tinker et al., 2016) have documented the extensive occurrence of stranded sea otters that bear bite wounds from white sharks, but have not (even partially) been consumed. Beyond this, an extensive study of white shark stomach contents (Klimley, 1985) revealed zero sea otter parts. Further experimental trials have revealed that white sharks selectively consume high‐calorie blubber tissues (Pratt, 1982) that sea otters lack, and overwhelmingly reject items that are not established prey (99.9% rejection rate, Hammerschlag, Martin, Fallows, Collier, & Lawrence, 2012).

Even though white shark bites to otters therefore appear exploratory, Tinker et al. (2016) documented a steep and related increase in sea otter mortality. Borrowing from other studies (e.g., Lowe et al., 2012), Tinker et al. (2016) suggested that white shark population growth, and particularly increases in the juvenile stage class, may be a factor. Though these white shark population changes have not been rigorously demonstrated, such growth may be explained in a few ways. First, reduced juvenile mortality due to the California gillnet ban of 1994 (Lowe et al., 2012) likely has encouraged stronger juvenile cohorts by boosting juvenile recruitment. Second, as immature sharks have no social learning (e.g., Fujii, McLeish, Brooks, Gaskell, & Houtan, 2018), and are less experienced in distinguishing targets, juvenile white sharks may simply target the wrong surface prey. Another explanation for the increased shark‐bite mortality in otters is the population growth of white shark prey (pinnipeds, cetaceans), and a corresponding population increase in white sharks.

The geographic distribution of shark‐bitten otters may be important. Most strandings of shark‐bitten otters occur in sandy, shallow habitats near the northern and southern range peripheries (Nicholson et al., 2018; Tinker et al., 2016). A recent analysis showed that shark bites occur almost exclusively where *Macrocystis* and *Nereocystis* kelp canopies cover <10% of the available habitat (Nicholson et al., 2018). This makes sense as kelp forests provide shelter for sea otters, potentially protecting them from visual ambush while they rest at the ocean surface. Besides lacking kelp, the range peripheries also contain two major elephant seal colonies. Año Nuevo Island (37.108°N, 122.338°W) and adjacent areas host 2,000 elephant seal births annually (Le Boeuf & Laws, 1994) with Point Piedras Blancas (35.666°N, 121.284°W) producing over 4,000 (Lowry et al., 2014) each year. Beyond sea otters, white sharks also mistarget humans as prey. However, in contrast with otters, the rate of human shark bites over the past 50 years has increased only marginally and per‐capita risk has decreased by 90% (Ferretti, Jorgensen, Chapple, Leo, & Micheli, 2015).

Though it might not have been significant historically, several factors indicate that white sharks may be currently limiting the recovery of California sea otters. The sea otter population is not spatially homogeneous, but densest at the range center on the Monterey Peninsula (Nicholson et al., 2018). There it is either approaching, or already at, carrying capacity (Gagne et al., 2018; Smith, Newsome, Estes, & Tinker, 2015). As a result, any appreciable and sustained population growth must come from the range peripheries. Yet, it is in these peripheries where a high rate of injurious and fatal interactions with white sharks appears to be stalling population growth (Tinker et al., 2016). Expanding the sea otter distribution beyond these shark dominated peripheries—perhaps through surrogacy‐assisted reintroduction (Mayer et al., [Link])—is one proposed solution. However, such interventions may not solve the problem. Therefore, we seek a better understanding of the interactions between white sharks and otters as the underlying causes can inform ecosystem conservation during a time of dramatic ecosystem changes (Di Lorenzo & Mantua, 2016).

Here, we integrate independent data streams (Ferretti et al., 2015; Jorgensen et al., 2019; Nicholson et al., 2018; Tinker et al., 2016) to more cohesively explain the observed patterns of shark–otter interactions and to inform sea otter as well as ecosystem conservation. We hypothesized seasonal trends in shark‐bitten otters would mirror trends in mistargeted shark prey, expecting that they will be contextualized by prey foraging trends but not directly coincident with this targeted feeding season. Furthermore, as shark–otter interactions are greatest at the range peripheries, we expected demographic implications that may limit natural dispersal and range expansion. As a result, we investigated sex and age biases that indicated heightened shark bite risk for demographic classes that are more vagile and explorative. Next, as multiple white shark demographics may be involved—each subject to different life histories, ecological mechanisms and different growth rates—we predicted the seasonality of shark–otter interactions may not be constant through time. Instead, we expected more recent strandings to encapsulate broader portions of the year, including months when low coastal densities of adult white sharks are expected due to migratory habits (e.g., spring; Figure 2 in Carlisle et al., 2012). Finally, due to the emerging significance of white shark bites to otters and other predator impacts (Jorgensen et al., 2019), we anticipated that the traditional sea otter trophic model may need revision. Collectively, we hope the integrated data and analyses here generate new insights into protected species population recovery and whole ecosystem restoration.

## 2 METHODS

### 2.1 Predator–prey versus non‐trophic interactions

Long‐term population monitoring and tagging studies have produced robust data streams on the seasonal presence of adult white sharks and elephant seals along the central coast of California (Brown et al., 2010; Jorgensen et al., 2019, 2012). We summarized phenological data on coastal shark visitation to mainland and island monitoring sites (Southeast Farallon Islands, Tomales Point, Año Nuevo) from published acoustic tagging studies with continuous monitoring spanning multiple years (Jorgensen et al., 2019, 2010). These data (archived at https://osf.io/b7su4/?view_only=f4f874c19e044ea5951a9ac355954d9f) were aggregated as number of individuals detected per day across all sites monitored (Southeast Farallon Island, 37.69 N, −123.00 W; Tomales Point, 38.24 N, −123.00 W; and Año Nuevo Island, 37.11 N, −122.34 W) from 2007 to 2013. We summarized a similar phenology for juvenile elephant seal abundance based on beach counts on Southeast Farallon Island from 1987 to 2013 (Jorgensen et al., 2019). Seal data, produced from weekly comprehensive island census surveys, were specific to Southeast Farallon Island haul outs, but closely resembled previous literature on elephant seal phenology at other sites (e.g., Año Nuevo, Le Boeuf & Laws, 1994). Seasonal count biases were minimized by exhaustive sampling methodologies. Pinniped censuses were conducted weekly since 1987, while listening buoys monitoring tagged shark detections were deployed for multiple years and downloaded annually (Jorgensen et al., 2019).

We transformed all counts into relative density through time (Jorgensen et al., 2019, 2010; Robinson et al., 2012). For both shark and seal data, phenological density was estimated from kernel smoothing of total number of individuals detected on a given day throughout the monitoring period. Cumulative distribution functions (CDFs) describe the temporal distribution of prey and non‐prey interactions to quantify connections between the two dynamics.

During coastal behavioral phases, sharks bite non‐prey species including both humans and sea otters (Ames & Morejohn, 1980; Curtis et al., 2012; Tricas & McCosker, 1984). We extracted human shark bite incidents from the Global Shark Attack File (available at https://www.sharkattackfile.net/incidentlog.htm) a public database cataloguing shark–human interactions. Following Ferretti et al. (2015), we restricted the analysis to California cases, where white shark interactions were unprovoked and injurious (1997–2018, *n* = 75). Phenological density was estimated from kernel smoothing over the seasonal period when human shark bites were observed.

We extracted observations of shark‐inflicted trauma from the record of California sea otter strandings. From 1997 to 2017, members of the sea otter stranding network, led by the Monterey Bay Aquarium, responded and recovered live‐stranded sea otters along their entire range. In each stranding (*n* = 431) collection efforts recorded metadata on the event (e.g., date, location), animal (e.g., morphometrics, sex, age class), and apparent cause of stranding (Nicholson et al., 2018). Shark trauma was assessed and assigned as the cause of stranding on the basis of visible wounds, lacerations, and injuries and the absence of other stranding symptoms (see https://osf.io/vfz54/, Table 1). Using this repository data, we created annual summaries of shark bitten strandings by age and by sex. By focusing on live‐stranded animals, we constrained our analyses to animals without postmortem decay, and whose symptoms and trauma are more diagnostically and rigorously determined (Nicholson et al., 2018).

**Table 1:** Live strandings structured by sex, maturity, and shark‐attribution through years. Frequency and proportion of shark‐ and non‐shark‐attributed live strandings are tallied for 3‐year periods, starting in 1997

Totals		Proportion shark bitten	Years	Sex	Mature		Immature	
Shark	No shark				Shark	No shark	Shark	No shark
4	12	0.33	1997–1999	Male	0	3	0	3
				Female	2	3	2	3
4	30	0.13	2000–2002	Male	2	5	1	7
				Female	1	10	1	8
7	48	0.15	2003–2005	Male	4	12	1	8
				Female	1	21	1	7
7	58	0.12	2006–2008	Male	4	9	2	17
				Female	7	13	0	19
23	39	0.59	2009–20011	Male	9	10	3	9
				Female	3	14	4	6
33	49	0.67	2012–2014	Male	19	13	4	10
				Female	1	17	7	9
47	70	0.67	2015–2017	Male	20	15	17	17
				Female	1	15	9	23

### 2.2 Demographic analyses and seasonal expansion

Given the geographic bias of shark‐bitten otters to kelp‐sparse regions (Nicholson et al., 2018), we assessed whether highly vagile demographics were a significant risk factor for shark bites. Following Nicholson et al. (2018), we classified otters into the following stages: immature, nonbreeding individuals (<4 years old), and mature adults (>4 years). We further excluded pup (<6 months old) and non‐shark‐bitten geriatric (>10 years) strandings as their stranding circumstances are unique to their demographic. To achieve adequate sample sizes and provide more robust assessment of sample variability, we resampled the observed data within 3‐year bins through nonparametric bootstrapping (*n* = 1,000 per 3‐year bin). Next, we used chi‐squared tests of independence to examine the demographic tendencies of shark‐bitten otters. All statistical and quantitative analyses were conducted in the R environment (R Development Core Team, 2018) with tidyverse (Wickham et al. 2017) and broom (Robinson et al., 2018) packages. In each replicate, we quantified the compositional metric for each resampled cohort, including calculating age/sex composition, male bias, and seasonal intervals.

To test whether the season of shark–otter interactions expanded over time, we used bootstrapped data to quantify temporal clustering in the data. To fully incorporate the temporal variability within the resampling, we implemented a Bayesian procedure to determine a realistic, but not identical temporal distribution of stranding events. Within each resampled stranding event, an estimation was made for an adjusted stranding date using a truncated normal prior. This prior distribution was centered on the date of the actual stranding observation, while fitting the variability to the median absolute deviation of dates observed within the replicated time bin. This variability was scaled according to each bin's observed number of strandings, so that bins with numerous shark‐bitten strandings were more temporally‐constrained to observed data in the bootstrap estimation. By fully incorporating the observed temporal variability, the resampling procedure could produce realistic temporal distributions of shark‐bitten strandings without oversampling exact dates in the observed data. We calculated the core shark bite season from the core 80% interval of each resampled distribution.

## 3 RESULTS

**Figure 1:**
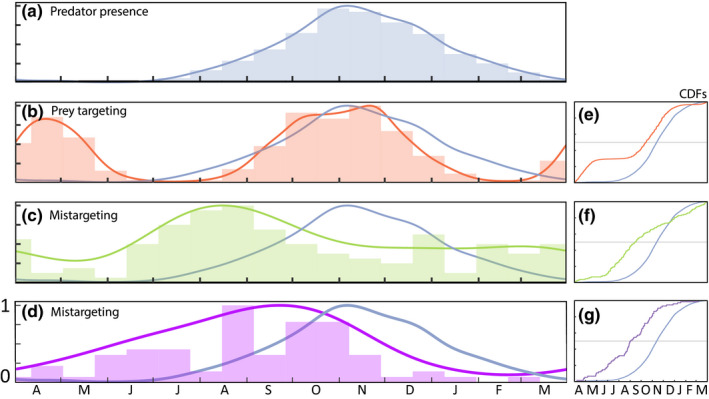
Mistargeted white shark interactions peak before their actual foraging season. Coastal activity of adult and subadult white sharks (blue) follows a distinct seasonal phenology (a) that matches fall aggregations of the shark's preferred prey, immature elephant seals (b). Phenologies of shark‐inflicted trauma on non‐prey species (c, d), including otters (c) and humans (d), peak prior to the onset of the shark's foraging season on prey seals. Temporal densities are fit to each dataset to describe the annual phenology (a–d). Cumulative distribution functions are fit (e–g) to each dataset to quantify their interaction with the coastal pulse in adult sharks

From 1997 to 2017, sea otter strandings attributed to incidental shark bites peaked seasonally in late summer (Figure 1). This peak preceded the shark's focused foraging activity evident in the direct overlap between coastal white shark presence (Figure 1a) and peak fall elephant seal haul out (Figure 1b,e). The timing of maximum shark–otter interactions coincided with a migratory transition period when adult white sharks seasonally return from offshore habitats (Figure 1c,f). During this annual transition, a similar seasonal pulse occurred in shark–human interactions in California from 1950 to 2017 (Figure 1d). Trends in both non‐prey interactions decreased over the fall white shark foraging season (Figure 1c,d), when shark predation on pinnipeds increased (Brown et al., 2010). Bites on humans diminished in the fall (Figure 1g). Shark‐bitten otter strandings also diminished but then persisted at moderate levels through the winter when sharks and seals had dispersed (Figure 1c). Importantly however, otters are found in water year‐round, whereas human use of the nearshore marine environment decreases significantly following summer months (Ferretti et al., 2015).

**Figure 2:**
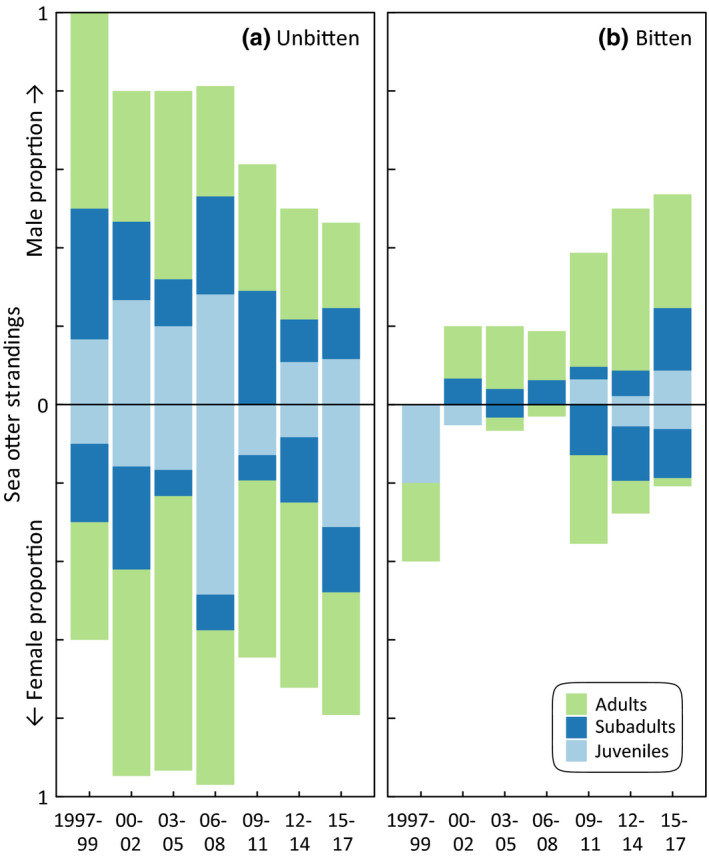
Shark bites increasingly threaten male sea otters. Demographic proportions of stranded males (upper panels a, b) demonstrate unique shark‐bite threats for mature males (b, green) compared to other stranding causes (a). Females strand primarily due to other causes (lower panels a,b), though the incidence of shark‐bite trauma in immature classes (blue) is increasing (b)

Increases in shark‐bitten otters did not affect age and demographic classes evenly. Instead, stranded males and immature individuals were most likely to exhibit shark‐inflicted wounds. Shark trauma comprised increasing proportions of the overall live stranding record (Table 1, Figure 2). The ratio of males to females was significantly higher among strandings attributed to shark trauma compared with those with other causes (*χ* ^2^ = 19.04, *df* = 1, *p* < 0.0001, Table 1). Across sexes, strandings with shark bites comprised relatively even proportions of mature and immature otters (*χ* ^2^ = 1.100, *df* = 1, *p* = 0.29, Table 1). Among males with shark bites, adults stranded more frequently than immatures relative to other stranding causes (*χ* ^2^ = 6.92, *df* = 1, *p* < 0.01, Table 1). However, among females, this same ratio was marginally insignificant (*χ* ^2^ = 2.98, *df* = 1, *p* = 0.08, Table 1). Overall, there was an increase in immature female otters stranding with shark bites (Figure 2). In the past 10 years, a greater proportion of immature females stranded with shark bites compared with prior data (*χ* ^2^ = 4.25, *df* = 1, *p* < 0.04, Table 1). Similarly, since 2008, immature male otters exhibited an increase in the proportion of shark‐bitten strandings (*χ* ^2^ = 7.63, *df* = 1, *p* < 0.005, Table 1).

**Figure 3:**
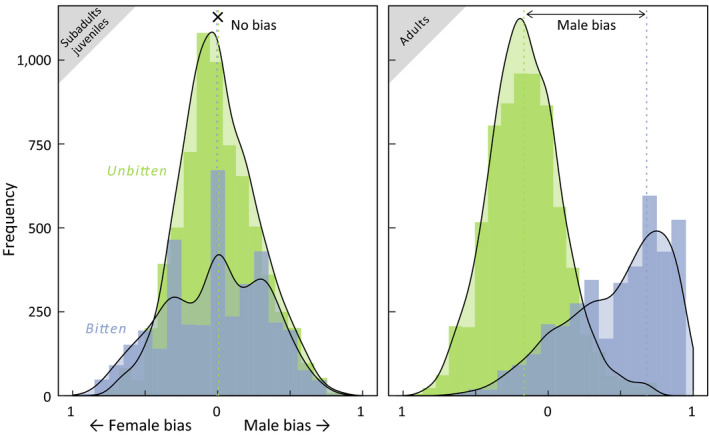
Adult male otters are at greatest risk of shark bites. Bootstrap resampling demonstrates the age‐based disparity in sex ratios between shark‐bite strandings (blue) and other causes (green). Shark‐bitten strandings of immature individuals exhibit relatively even probability of being male or female, in comparison to the heavily male‐biased rates observed in adult age classes

Adult otter strandings by shark bite were significantly male‐biased (Wilcox text, W = 7, *p* < 0.03, Table 1) in comparison to other stranding causes (Figure 3, Table 1). Notably, this difference was absent for immature otters (Figure 3). In adults, there was a 36.6% greater probability of a male stranding due to shark bite (Figure 3, male bias: median = 0.78; 95% CI 0.608–0.952) compared with other stranding causes (male bias median = 0.42, 95% CI 0.197–0.643). Sex bias was absent in younger age classes, with immature shark bite strandings exhibiting even sex ratios (male bias median = 0.50, 95% CI 0.250–0.750). Importantly, within this immature age group, the sex ratio of shark bite strandings was indistinguishable from the sex ratio in other stranding causes (Wilcox test, *W* = 27, *p* > 0.80).

**Figure 4:**
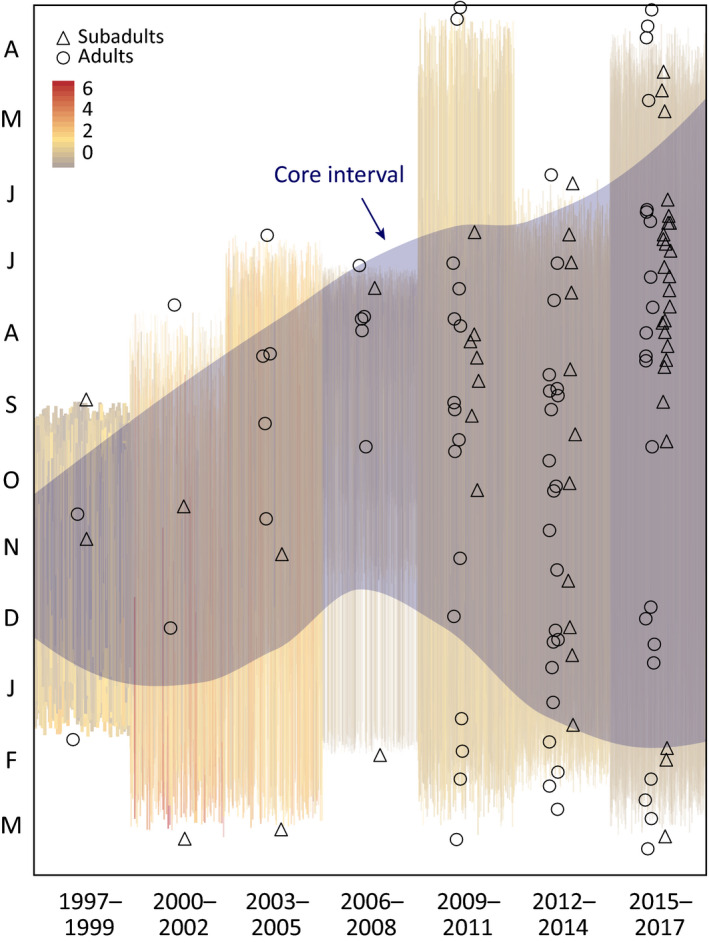
The threat of shark bites is advancing seasonally and expanding annually. Shark‐bitten strandings of mature (circles) and immature (triangles) otters occurred more broadly throughout the year, especially earlier during summer months. Resampling (bars) showed temporal variability in the core 80th percentile encapsulating the seasonal range during which shark‐bitten otters are observed. Now observed throughout the entire year, apparent loss of any phenological refuge from shark bites can negatively affect otter recovery processes. Notably, slight gaps are observed during the shark's peak fall foraging season

Seasonal trends in shark‐bitten otter strandings over the study period broadened from a few months to encompass much of the year. Figure 4 depicts the expansion of the seasonal envelope more recently broadening to include the entire summer and some of the spring. We observed this expansion for all age classes, while adult strandings contributed predominantly to winter expansion (open circles, Figure 4). A simple linear regression indicates that, over the past two decades, the median date for shark bites advanced by 82 days (*m* = −13.73 days triannually; *R* ^2^ = 0.287, *p* < 0.0001).

## 4 DISCUSSION

Species recovery efforts often aim to increase population growth rates and abundance, while decreasing threats, and promoting restoration throughout a species historical range (Seminoff et al., 2015). This is particularly true for sea otters, yet their recovery has been sluggish when compared to pinniped populations that exhibited maximal growth and full range expansion over shorter periods of protection (Laake, Lowry, DeLong, Melin, & Carretta, 2018; Lowry et al., 2014, 2017). Otter populations have increased from ~50 individuals in the early 1900s to a present‐day abundance of more than 3,100 individuals (Tinker & Hatfield, 2017), largely due to the elimination of direct exploitation and fishery entanglement (Estes, Hatfield, Ralls, & Ames, 2003). However, recolonization of their historical California range has stalled with no appreciable expansion in 20 years (Nicholson et al., 2018; Tinker & Hatfield, 2017). Inside the densely populated core range near the Monterey Peninsula, density‐dependent resource limitation affects survival, but at the kelp‐sparse range peripheries, shark interactions have unexpectedly become an emergent property and are driving sea otter stranding trends (Nicholson et al., 2018; Smith et al., 2015). Our analyses here inform these dynamics in several ways.

Shark–otter interactions align closely with other non‐prey interactions (Figure 1d), but precede the sharks' targeting of elephant seal prey (Figure 1b). Following the sharks' extended pelagic migratory phase, adult white sharks concentrate in central California near aggregations of juvenile elephant seals at coastal rookeries that crest from October–November (Brown et al., 2010; Pyle et al., 1996). In this period when white sharks are consistently detected at rookery sites, interactions with otters are comparatively less frequent. After 2004, shark‐bitten live strandings are virtually absent or infrequent during this focused foraging period (Figure 4). The underlying incentives during the migration‐foraging transition may be influential here (Benson et al., 2018; Lyons et al., 2013). Following a 3–6 week migration from distant oceanic habitat, adult sharks are likely resource depleted (Brown et al., 2010; Carlisle et al., 2012; Jorgensen et al., 2019) upon arriving at the coast. Then, sharks must navigate toward rookery locations on paths that increase their probability of encountering other coastal inhabitants. Previously, Tinker et al. (2016) hypothesized that naïve juvenile white sharks may be responsible for mistaking otters for more fat‐rich prey items. However, the link found here between the seasonal migratory movements of adult and subadult white sharks (Figure 1a) implicates these larger white sharks that are transitioning to coastal feeding stations. On the tail end of the foraging season, adult white sharks have high lipid reserves (Del Raye, Jorgensen, Krumhansl, Ezcurra, & Block, 2013), fewer foraging incentives, and there are (perhaps correspondingly) few shark–otter interactions (Figure 1c).

From 1997 to 2017, the seasonal span over which sharks interacted with otters quadrupled from 2 to 8 months (Figure 4). In particular, the annual onset of these interactions shifted earlier, agreeing with findings in dead strandings (Tinker et al., 2016). Tagging data indicate that virtually all mature individuals are offshore from April‐June (Carlisle et al., 2012; Jorgensen et al., 2010). Therefore, the recent (2015–2017; Figure 4) cluster of strandings during these spring months may be attributed to immature white sharks that do not yet venture offshore (Hoyos‐Padilla et al., 2016). The seasonal movements of this age class are not well known (Benson et al., 2018; Lyons et al., 2013), but such demographics generally show strong environmental forcing (e.g., Ascani, Houtan, Lorenzo, Polovina, & Jones, 2016) and this appears to hold for white sharks as they develop endothermy (Weng et al., 2007; White, 2016). Due to episodic ocean warming, the thermal habitat envelope for juvenile white sharks has extended seasonally north of Point Conception into Monterey Bay (White, 2016), to within the core of the sea otter distribution. As a result, recent and anticipated future warming may increase encounter rates between juvenile white sharks and sea otters.

Future work must address persistent knowledge gaps as they relate to sharks interacting with bitten otters. In particular, bite forensics (French et al., 2017) and possibly genetic tools (Fotedar, Lukehurst, Jackson, & Snow, 2019) could generate greater discernment regarding the sex, size, or age classes of sharks that interact with otters. Through greater ecological understanding, knowledge gains can better contextualize the novel emergence and recent acceleration of mistargeting otters by sharks. In particular, greater attention to the less‐studied feeding, movement, and habitat patterns of sharks transitioning from piscivory and southerly juvenile habitats to mammal feeding near pinniped colonies can elucidate the contribution of this development. Furthermore, detailed diet estimation of bitten otters can define risk factors of alternative foraging strategies (e.g., kelp‐focused versus sandy seafloor) while identifying depths or habitats prone to shark–otter interaction. Finally, predictive modeling using ecosystem variables (e.g., kelp cover, water temperature, storm intensity, and climatic indices.) can pinpoint environmental cues that influence interaction rates and integrate into population growth and expansion models (Tinker, Doak, & Estes, 2008) to set expectations for recovery. At this point, evidence points largely to range expansion as the critical element of continued recovery in otters.

Range expansion in otters is associated with pioneering behavior among males and immatures, the same demographics most frequently bitten by sharks. Mature female otters are more likely to reside in shallow kelp forest habitat (Tinker et al., 2008) during reproduction, lactation, and pup provisioning (Smith et al., 2015). The disproportionate impact of white sharks on male otters (Figures 2 and 3) has intensified risk for explorative foraging beyond kelp habitats by dispersing males (Smith et al., 2015; Tinker et al., 2008). Additionally, the broadening seasonal span of shark bite risk (Tinker et al., 2016) could constrain the window when safe dispersal into novel habitats (Lafferty & Tinker, 2014; Tinker et al., 2008) is possible. As such pioneering behavior is foundational to new establishment (Lafferty & Tinker, 2014; Tinker et al., 2017), the demographic biases and broadening seasonal trend observed in shark‐bitten otters could inhibit range expansion and overall population growth (Lafferty & Tinker, 2014; Tinker et al., 2017; USFWS, 2015).

**Figure 5:**
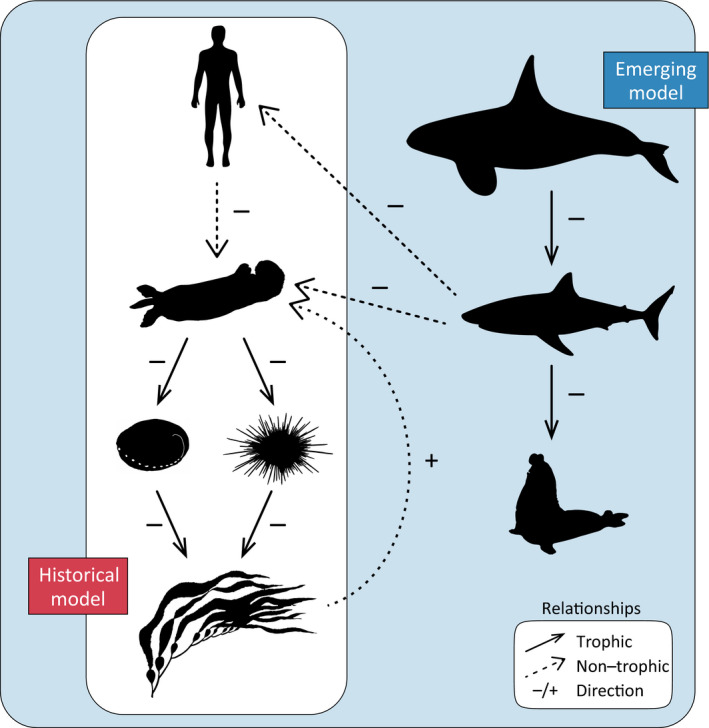
An emerging model of sea otter dynamics in California kelp forest ecosystems. Multiple decades of empirical study have demonstrated the important role that Southern sea otters play in regulating kelp forest ecosystems. By foraging on invertebrate herbivores, like abalone and spiny urchins, they keep kelp herbivory in check and allow kelp forests ecosystems to thrive. Historical threats to this model were the hunting of otters for the fur trade, and after this, fishery gear entanglement and boat strikes. Though these anthropogenic stressors have largely been removed, the sea otter population growth has remained stagnant. The emerging model we document here, of non‐trophic, but often fatal bites from white sharks may help explain this. Where the historical model largely supports the narrative that otters are critical for kelp, the emerging model also supports the reverse narrative that kelp may be critical for otters. Emerging influences from killer whales to white sharks, and white sharks to otters have recently emerged as significant. Solid lines represent trophic interactions, dashed lines represent non‐trophic interactions, and signs indicate the direction of effect

It has been established that sea otters are a keystone species, engineering the growth and stability of kelp ecosystems through trophic cascades (Estes & Duggins, 1995) (Figure 5). In recent years, California has experienced a general decrease in kelp cover associated with environmental anomalies (Edwards, 2004; Foster et al., 2013; Graham, Halpern, & Carr, 2008) and disease‐related die‐offs of key species (Hewson et al., 2014), particularly outside of the core otter range. An expansion of the core otter range could potentially mitigate this ecosystem trend in recolonized areas. However, shark bites present a compounding problem because of elevated bite risk outside of kelp habitat (Nicholson et al., 2018), while at the same time disproportionately impacting pioneering individuals that initiate kelp growth outside core areas (Figures 2 and 3). As such, it may be challenging for otters to gain a foothold in kelp‐free range periphery areas, even where otters and kelp have thrived historically. Continued rehabilitation efforts (Mayer et al., [Link]) and targeted releases that promote occupancy in alternate habitats, such as estuaries (Hughes et al., 2016; Mayer et al., [Link]), may be the most effective approach for perpetuating otter recovery in range peripheries. The role of otters in promoting kelp forest communities has been documented and now may be more significant than ever. Here, however, we provide evidence of the unexpected feedback where a keystone species can provide for its own refuge habitat (Figure 5), which can increase connectivity particularly at the peripheries of its population range.

Our analyses here build upon previous studies and help narrate some of the ecosystem challenges in restoring the California sea otter population. As a foundational species, kelp is a living substrate that forms a physical habitat and is the existential base of a complicated food web (Steneck et al., 2002). Multiple stressors currently threaten the persistence of kelp forests across the coastal waters of California. In the south severe storms and marine heat waves (Edwards, 2004; Foster et al., 2013; Graham et al., 2008) are influential, and in the north disease‐related die‐offs (Hewson et al., 2014) are creating extensive barrens. Against these bottom‐up, and largely climate‐driven processes, is the historical understanding that California kelp forests are regulated through a top‐down cascade that originates with sea otters (Estes & Duggins, 1995). Tinker et al. (2016) advanced this simple trophic model by highlighting the dramatic increases in shark‐related mortality and the population influence to sea otters. Here, we expand on their analysis by demonstrating that these interactions have a pronounced phenology (Figure 1d) that is strikingly similar to the phenology Ferretti et al. (2015) documented for mistargeting of humans as prey by white sharks (Figure 1c). Beyond the basic phenology, we further quantify that the seasonal refuge sea otters may have from shark bites is shrinking (Figure 4). Where previous studies documented that the edges of the sea otter range are hotspots for shark bites (Nicholson et al., 2018; Tinker et al., 2016), we show that these interactions are disproportionately targeting the pioneering sea otter demographic that would contribute the most to expanding the sea otter distribution (Figure 3). Lastly, we integrate the positive bottom‐up influence of kelp (Nicholson et al., 2018) and the indirect and positive top‐down influence of orcas (Jorgensen et al., 2019) into a cohesive conceptual ecosystem model for California sea otters (Figure 5). Resolving such complexities, through monitoring multiple species at multiple trophic levels, may become increasingly influential in the success of both recovering protected species and the ecosystems in which they all live.

## CONFLICT OF INTEREST

None declared.

## AUTHOR CONTRIBUTIONS

JM, SJJ, KSV and TN designed the study. JM, SJJ and KSV analyzed the data and drafted the figures. JM, SJJ and KSV wrote the manuscript. All authors contributed data and edited the manuscript.

## Data Availability

All data used in this study are from published datasets and studies fully referenced in the main text.
